# Comparative Analysis of Adhesive Retention and Denture Weight in Different Residual Ridge Morphologies: A Cross‐Over Randomized‐Controlled Trial

**DOI:** 10.1002/cre2.70118

**Published:** 2025-03-24

**Authors:** Naseer Ahmed, Maria Shakoor Abbasi, Asra Salahuddin, Lareb Tariq, Sarrah Siraj, Gotam Das, Ghazala Suleman, Fahim Vohra, Artak Heboyan

**Affiliations:** ^1^ Department of Prosthodontics Altamash Institute of Dental Medicine Karachi Pakistan; ^2^ Department of Prosthodontics, School of Dentistry Shaheed Zulfiqar Ali Bhutto Medical University Islamabad Pakistan; ^3^ Department of Prosthodontics, College of Dentistry King Khalid University Abha Saudi Arabia; ^4^ Prosthetic Dental Science Department, College of Dentistry King Saud University Riyadh Saudi Arabia; ^5^ Department of Research Analytics, Saveetha Dental College and Hospitals Saveetha Institute of Medical and Technical Sciences Saveetha University Chennai India; ^6^ Department of Prosthodontics, Faculty of Stomatology Yerevan State Medical University after Mkhitar Heratsi Yerevan Armenia; ^7^ Department of Prosthodontics, School of Dentistry Tehran University of Medical Sciences Tehran Iran

**Keywords:** alveolar ridge morphology, biomechanics, complete dentures, denture adhesives, denture weight, resorbed ridges, retention

## Abstract

**Aim:**

The aim of this study was to compare the retentive strengths of various forms of denture adhesives (paste, powder, and strips) on different types of mandibular residual alveolar ridges, considering their respective denture weights.

**Materials and Methods:**

In this crossover randomized‐controlled trial, the patients were randomly and equally divided into 3 groups based on clinical features and radiographic findings according to the Wical–Swoope classification. Three forms of denture adhesives were used, including powder, cream, and strips, for three residual ridge types. Quantification of retention without adhesive was carried out as a control. The retentive strength of adhesives was compared in each ridge pattern and correlated with the denture weight. The study was registered at https://clinicaltrials.gov (identifier number: NCT05063422).

**Results:**

The mean retentive strengths of dentures without adhesive (control) were relatively low across all ridge classifications, ranging from 0.27 to 0.69 lb. In contrast, the experimental groups utilizing Fittydent and Polygrip adhesive products showed significantly improved retentive strengths, with Fittydent cream and Polygrip cream showed the highest enhancement, ranging from 1.01 to 2.57 lb across different ridge types. Furthermore, significant mean differences were observed between ridge classes and the retentive strength of each adhesive product.

**Conclusion:**

The study highlights the crucial role of tailored denture adhesive selection in optimizing retention for denture wearers. Polygrip cream demonstrated superior effectiveness across various ridge classifications compared to Fittydent cream, powder, and strips. These findings emphasize the importance of personalized treatment approaches based on ridge types and denture weight.

## 1 Introduction

A denture is considered a viable treatment for edentulism and significantly affects the quality of life for millions of individuals globally (Chebib et al. 2022). A crucial factor in a successful denture is its retention, which refers to the ability of the denture to resist dislodging forces during function and speech (Chebib et al. 2022). Denture adhesives play a pivotal role in enhancing the stability and retention of removable dentures, thereby improving comfort and quality of life for denture wearers. Despite advancements in dental technology, selecting the most suitable adhesive remains a challenge, particularly considering the diverse anatomical variations among denture wearers (Kurogi et al. 2023).

The efficacy of denture adhesives in enhancing denture retention and stability has long been a subject of interest in prosthodontic research (Giok et al. 2024). Historically, denture adhesives have been utilized primarily to address issues related to poor denture fit and compromised chewing efficiency. Early formulations often relied on simple adhesive materials such as gum Arabic or beeswax, offering limited benefits and inconsistent outcomes (Papadiochou et al. 2015). However, advancements in dental materials science have led to the development of modern denture adhesives, which leverage sophisticated polymers and adhesive technologies to provide superior retention and comfort for denture wearers (Nagaraj et al. 2021). Denture adhesives could significantly improve denture stability, masticatory function, and overall quality of life among edentulous patients (Klukowska et al. 2024; Darshini et al. 2022).

However, the effectiveness of denture adhesives is not universal across all clinical scenarios, as the anatomical characteristics of the residual ridge play a crucial role in determining adhesive performance (Maria et al. 2023). The classification of residual ridges into different categories based on their anatomical features, such as height, width, and mucosal quality, provides valuable considerations into the challenges associated with denture retention (Maria et al. 2023). In this regard, previous studies have emphasized the importance of considering ridge classification in denture treatment planning, highlighting the need for tailored approaches to denture adhesive selection (Ohwada et al. 2020; Shu et al. 2021).

Denture adhesives play a crucial role in enhancing the retention and stability of complete dentures, particularly in individuals with compromised residual ridge morphology (Giok et al. 2024). To serve this purpose, various adhesive products are available, including cream, powder, and strip formulations, each with unique properties and application methods. Fittydent and Polygrip are among the commonly utilized adhesive brands, offering diverse options to meet the specific needs of denture wearers. Fittydent adhesive products, known for their strong adhesion and long‐lasting hold, provide enhanced retention without compromising oral tissue health (Shu et al. 2021). On the other hand, Polygrip adhesives are reported to offer both adhesive strength and a cushioning effect to improve denture stability and comfort (Manes et al. 2011). Additionally, adhesive strips have gained popularity due to their ease of application and discrete nature, providing reliable adhesion without the mess associated with cream or powders (Papadiochou et al. 2015). Although these adhesive systems have demonstrated efficacy in enhancing denture retention, their comparative performance across different residual ridge morphologies remains an area of ongoing research. Understanding the advantages and limitations of each adhesive type is essential for optimizing denture retention and improving the quality of life for denture wearers.

An often‐overlooked factor in denture retention is the weight of the denture itself. Heavier dentures may benefit more from adhesive applications due to the increased forces that need to be counteracted to maintain retention (Johnson et al. 2013). Studies have shown that denture weight can significantly influence overall retention and stability, especially in mandibular dentures, where gravitational forces play a more prominent role (Rathi et al. 2020). Consequently, considering the weight of the denture in conjunction with the adhesive type and ridge morphology can lead to more effective and personalized treatment plans for denture wearers.

This study aimed to compare the retentive strengths of different forms of denture adhesives (paste, powder, and strips) on different forms of mandibular residual alveolar ridges. By explaining the associations between adhesive types and ridge morphology, this study seeks to provide valuable insights for dental practitioners to inform personalized treatment strategies and improve the quality of care for denture patients. This study hypothesized that the effectiveness of denture adhesives, specifically Fittydent and Polygrip in cream, powder, and strip formulations, varies significantly across different ridge types. Additionally, we predicted that mandibular denture weight correlates with adhesive efficacy, suggesting that heavier dentures may show increased retention with adhesive application.

## 2 Methodology

### 2.1 General Information

A cross‐over randomized controlled trial was conducted at the University for a duration of 2 years. The study was registered at https://clinicaltrials.gov; first submitted date: June 24, 2021; first posted and registered October 1, 2021; identifier number: NCT05063422.

The sample size was calculated using the WHO sample size calculator, considering a mean value of 1095.17 ± 668.64 (Manes et al. 2011) grams for Fittydent adhesive. Power of test β = 80. The confidence interval is 95%, and the margin of error is 5%. A sample size of 60 patients in total was calculated.

### 2.2 Screening and Initial Assessment

Sixty patients were recruited from the outpatient department of Prosthodontics at Altamash Institute of Dental Medicine. Patients visiting the department for complete denture treatment were screened. A preliminary assessment, including a thorough assessment of history and clinical examination, was conducted to determine eligibility.

### 2.3 Inclusion Criteria

- Age between 40 and 70 years.
- Completely edentulous mandibular arch.
- Patients willing to undergo new complete denture treatment.
- Committed to attending regular follow‐ups.

### 2.4 Exclusion Criteria

- Presence of unhealed extraction sockets.
- History of xerostomia or past use of ill‐fitting dentures.
- Patients with neuromuscular disorders, debilitating diseases, or immunocompromised conditions.
- Tobacco smokers and patients who required tissue conditioners or metallic base dentures.

### 2.5 Ethics Approval and Consent to Participate

Ethical approval was obtained from the Ethical review committee of the University. The patients provided informed consent before any interventions or data collection. Participation was voluntary, with risks and benefits outlined. Confidentiality was ensured, and it was explained to the patients that they could withdraw from the study at any point.

### 2.6 Grouping

**Figure 1:**
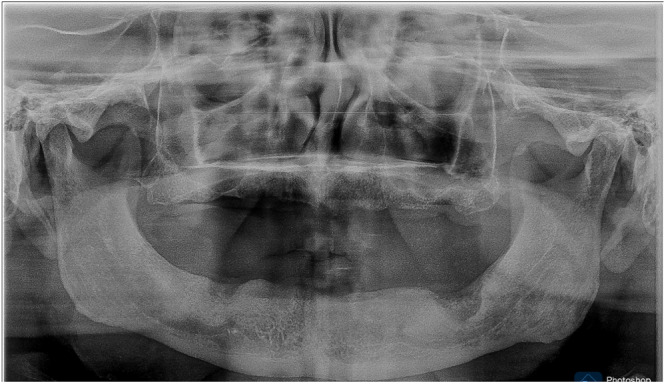
Class 1 ridge mild resorption with adequate bone support.

**Figure 2:**
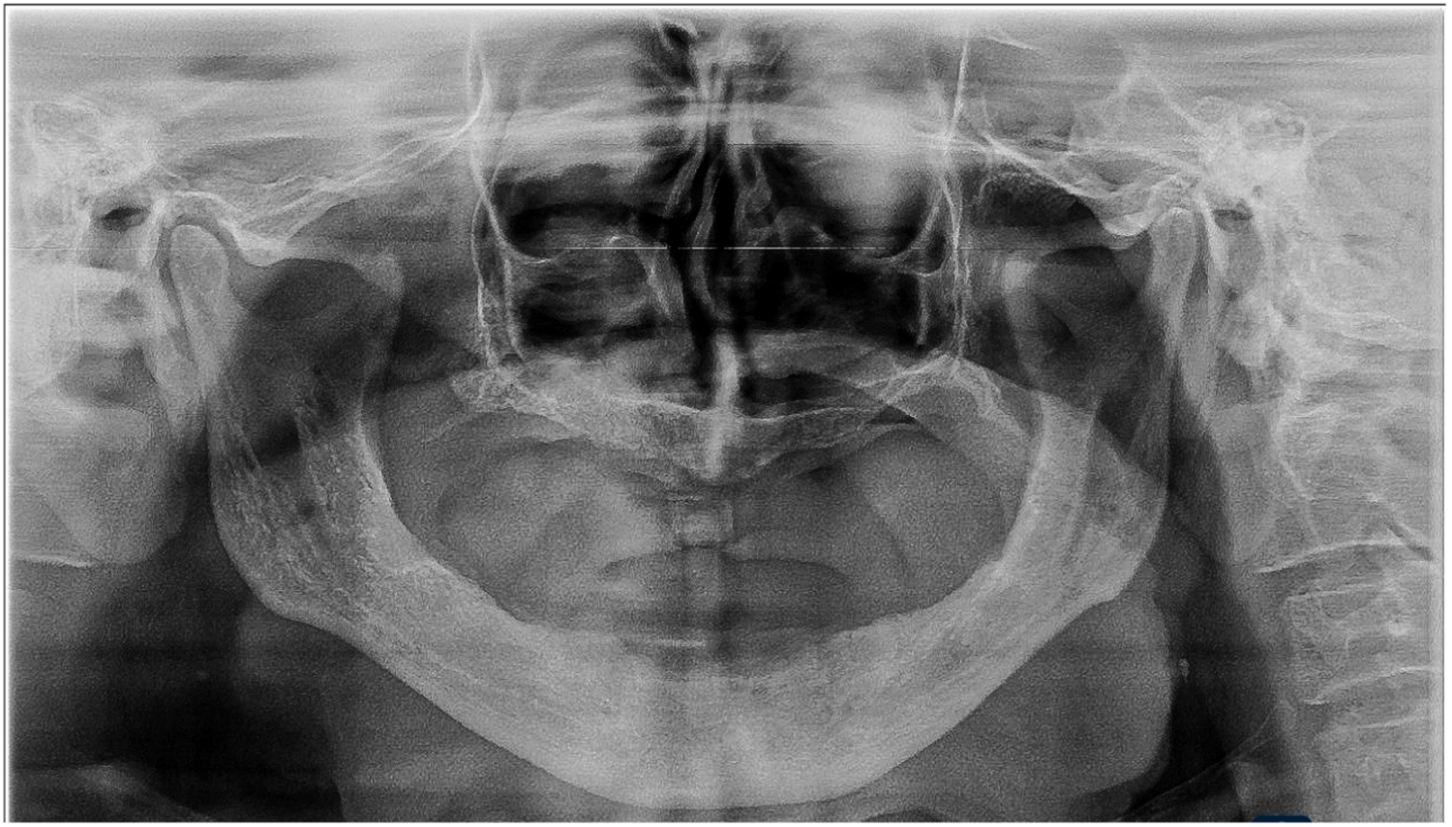
Class 2 ridge moderate resorption with reduced bone height.

**Figure 3:**
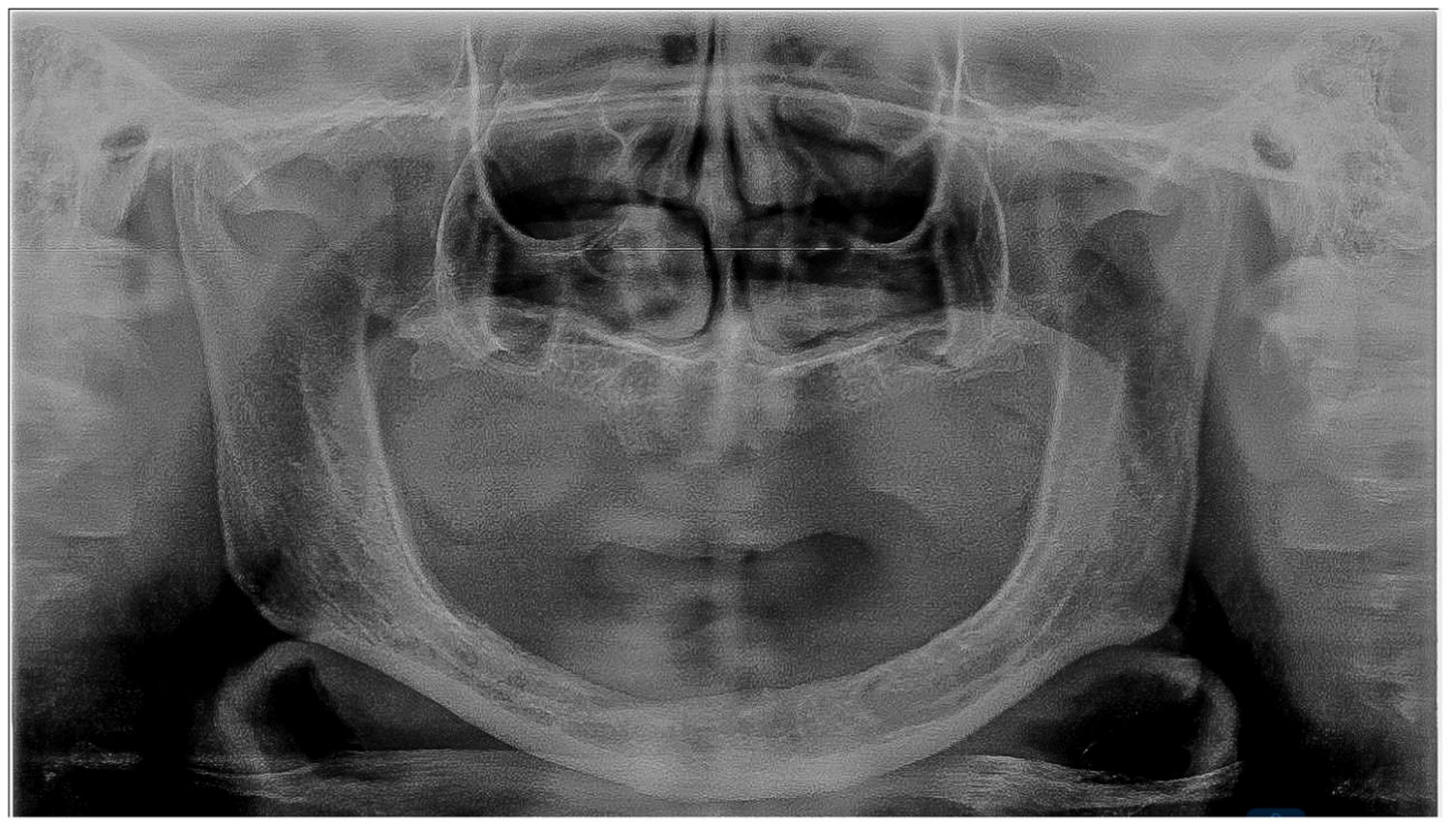
Class 3 ridge severe resorption with minimal bone support.

**Figure 4:**
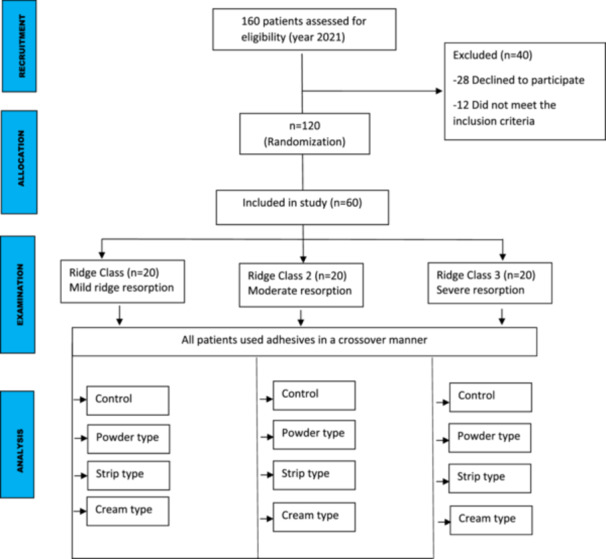
Consort flow diagram of the study.

All the patients underwent a complete assessment of history and oral examination after obtaining written informed consent. The selected patients were randomly and equally divided into three groups based on clinical features and radiographic findings according to the Wical–Swoope (Winkler 2013) classification. In panoramic radiography, if the space between the lower edge of the mandible and the lower edge of the mental foramen has been measured and then this is multiplied by three, the multiplication will yield a reliable estimate of the original height of the alveolar crest. Therefore, if there was a loss of up to 1/3 of the original vertical height, it was classified as mild resorption (Class 1). If the loss was from 1/3 to 2/3 of the original vertical height, it was classified as moderate resorption (Class 2), and finally, if the loss was 2/3 or more of the original vertical height, it was classified as severe resorption (Class 3) (Table 1, Figures 1, 2, 3). We adopted the CONSORT (Schulz et al. 2010) reporting guidelines in this study (Figure 4).

**Table 1:** Classification of ridge resorption based on the Wical–Swoope criteria.

**Ridge classification**	**Description**	**Grouping criteria**
Class 1	Well‐rounded ridge form with adequate height and width. Good bone support for dentures, leading to better stability and retention.	Loss of up to 1/3 of the original vertical height of the alveolar crest, indicating mild resorption.
Class 2	Moderately resorbed ridge with reduced height and width. Bone support is less than Class I, leading to moderate challenges in denture stability.	Loss of 1/3 to 2/3 of the original vertical height of the alveolar crest, indicating moderate resorption.
Class 3	Severely resorbed ridge with minimal height and width. Poor bone support, causing significant challenges in achieving denture stability and retention.	Loss of 2/3 or more of the original vertical height of the alveolar crest, indicating severe resorption.

### 2.7 Randomization and Allocation Concealment

To ensure unbiased assignment of participants to treatment groups, randomization was performed using computer‐generated random numbers by an independent researcher not directly involved in patient recruitment or data collection. The allocation sequence was generated before the start of the trial. Allocation concealment was achieved using the sequentially numbered, opaque, sealed envelopes (SNOSE) method. These envelopes contained the denture adhesive allocation and were opened sequentially only after participant enrollment, thereby ensuring that the allocation sequence was concealed from the outcome assessment investigators involved in patient recruitment and assignment. The allocation was carried out by a researcher not involved in outcome assessment or data analysis.

### 2.8 Blinding

Due to the nature of the interventions (different denture adhesive types), blinding of participants and dental assistants to the treatment was not feasible. However, the operators responsible for measuring denture retention strength were blinded to the type of denture adhesive used by each participant. This was achieved by ensuring that the denture adhesive application was performed by participants themselves without disclosing the type of adhesive to the assessors. Additionally, efforts were made to standardize the procedures for denture retention strength measurement to minimize potential measurement bias.

### 2.9 Denture Weight Measurement and Storage

A well‐constructed single acrylic complete denture was fabricated for the patients. The weight of the mandibular dentures was measured post‐construction, before any adhesive application or patient use. This initial measurement was conducted to establish a baseline weight of the dry denture. The dentures were carefully stored in a clean and dry environment to maintain their integrity until they were provided to the patients for use. The mandibular denture weight was measured using a digital jewelry weighing scale (Ming Heng Electronic Digital Scale‐MH‐999, Karnataka, India), calibrated before each measurement session to ensure accuracy and reliability. Calibration of the scale involved verifying its precision by using standard weights with known masses. This meticulous calibration process was performed to eliminate any potential sources of measurement error and to guarantee the scale's accuracy in providing precise weight measurements of the dentures. The occlusion of dentures was set as bilaterally balanced using semi‐anatomic teeth. After insertion, the post‐op complaints were resolved over frequent follow‐ups in a week. Before insertion and adhesive application for analysis, the fabricated dentures were stored in a standardized manner to maintain their integrity and ensure consistent conditions across all study participants. The dentures were kept in a clean and dry environment at room temperature, away from direct sunlight and moisture, to prevent any alterations in their properties. Additionally, the dentures were stored in individual labeled containers to avoid any potential cross‐contamination or damage during handling. This standardized storage protocol helped to preserve the dentures' characteristics and minimize any extraneous variables that could influence the analysis of adhesive efficacy.

### 2.10 Intervention

The intervention involved the application of Fittydent and Polygrip denture adhesive in cream, powder, and strip forms (Table 2), along with a control group on mandibular complete dentures for two consecutive days. The control utilized a saline solution (PLASALINE, 0.9% Sodium Chloride 20 ml, OTSUKA LIMITED, Pakistan). Participants were divided into three groups: powder‐type denture adhesive, cream‐type, and strips groups, with the control being the mandibular denture made for each class used without adhesive application. The co‐investigator provided instructions to the participants on how to apply the denture adhesives and saline solution. Each participant received guidance on the application methods for the denture adhesives.

**Table 2:** Denture adhesive types and manufacturer details.

Material	Type	Manufacturer	Composition
Poligrip Powder	Powder (Extra strength)	GlaxoSmithKline, Consumer Healthcare, Warren, NJ, USA	Cellulose gum, calcium/sodium, polyvinylmethylether maleic anhydride, copolymer, flavoring bagent
Poligrip Super	Cream (Original)	GlaxoSmithKline, Consumer Healthcare, Warren, NJ, USA	Methoxyethylene/maleic anhydride copolymer, petrolatum, cellulose gum, mineral oil, flavor
Poligrip Super	Strips (Comfort Seal)	GlaxoSmithKline, Consumer Healthcare, Warren, NJ, USA	Polyethylene glycol‐10000 (PEG‐90M), microcrystalline wax, polybutene, cellulose gum
Fittydent	Powder	FITTYDENT International GMBH, Pinkafeld, Austria	Sodium carboxymethylcellulose, starch, calcium/sodium, polyvinylmethylether maleic anhydride, colloidal silicon dioxide
Fittydent Super	Strips	FITTYDENT International GMBH, Pinkafeld, Austria	Polyvinylacetate, sodium carboxymethylcellulose, glycerintriacetate, petrolatum
Fittydent Super	Cream	FITTYDENT International GMBH, Pinkafeld, Austria	Polyvinylacetate, carboxymethylcellulose, petrolatum

In the powder‐type denture adhesive, dentures were cleaned, rinsed, and left wet. An appropriate amount of powder was then sprinkled onto the intaglio surfaces of the dentures, followed by shaking off the excess powder. Participants were instructed to press the dentures firmly and hold them briefly. They were advised to apply the denture adhesive before breakfast and dinner.

In the cream‐type denture adhesive, dentures were cleaned, rinsed, and dried and then, approximately 0.5–3.0 cm of cream was applied in 2–3 places. Participants were instructed to press the dentures firmly and hold them briefly. They were advised to apply the denture adhesive once a day before meals.

In the strip‐type adhesive, participants were instructed to apply it to the intaglio surfaces of the dentures once daily before breakfast. They were advised to clean and rinse the dentures thoroughly, leave them wet, and apply the strips according to the manufacturer's instructions, ensuring even coverage. The dentures were then pressed firmly to ensure proper adhesion. Participants were advised to reapply strips as needed if the adhesive effect diminished during the day and to remove any remaining strips before sleeping, reapplying new strips in the morning.

In the control, dentures were cleaned, rinsed, and left wet. Subsequently, 20 mL of saline solution was poured onto the intaglio surfaces of the dentures by squeezing the bottle to keep the surface wet. Participants pressed the dentures firmly and held them briefly. They were instructed to apply the saline solution before every meal.

Participants applied denture adhesives and saline solutions as instructed, adhering to the manufacturer's guidelines. They were also instructed to remove any remaining materials before sleeping at night and reapply new materials in the morning. Participants were permitted to apply additional denture adhesives and saline solutions as needed. Compliance with the intervention regarding the application of denture adhesives and saline solution was confirmed by measuring the weight of the remaining denture adhesives and counting the remaining number of bottles of saline solution at the end of the 2‐day intervention period (Kurogi et al. 2023).

### 2.11 Data Collection

**Figure 5:**
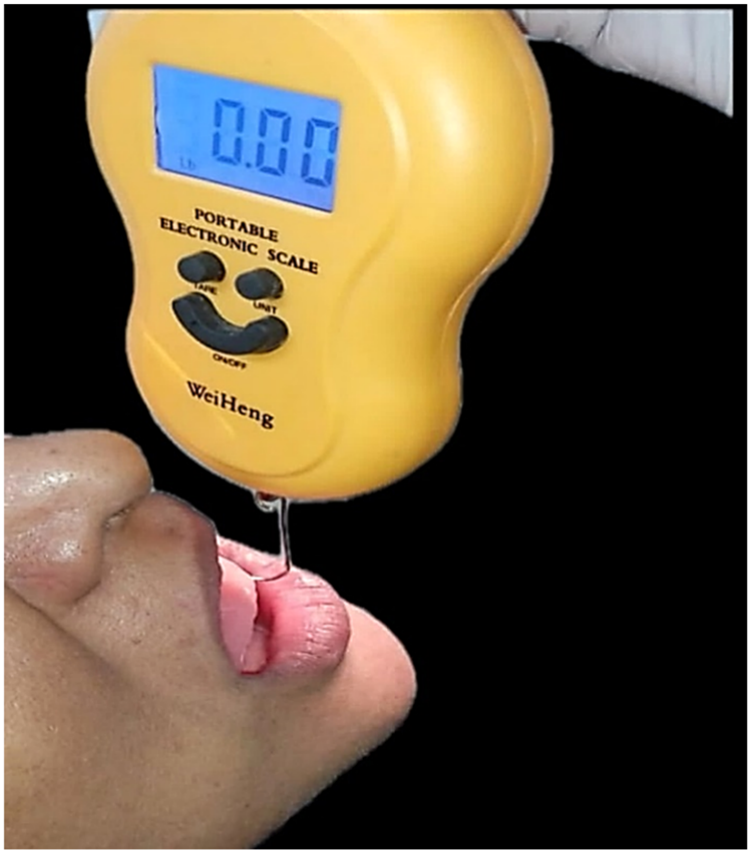
Spring scale used to record denture retention.

The outcome assessors, after the 2 days of follow‐up visit, recorded the baseline retention strength first as a control. A digital spring scale (WeiHeng; Zhejiang, China) was used to record the retentive strength readings in pounds (lb) (Figure 5). The dental adhesive retention tests were performed according to the International Standards Organization (ISO) 10873:2021 (International Organization for Standardization 2021) (Dentistry, Denture adhesives) recommendations. Before starting the procedure, the spring scale was calibrated, the second reading was taken using a (luggage hook) tension scale, and a comparison was performed. To reduce potential bias and ensure impartiality in our assessment, the readings were taken by three operators.

When dentures were placed in the mouths, patients were instructed to maintain maximum, non‐forced intercuspation for 5 min. After this, keeping the mouth open and the lower lips relaxed to avoid loss of peripheral seal, the tip of the spring scale was placed at the margin of the denture, in the recess of the lingual frenum. Traction force was then applied until the denture detached, the maximum retention force being recorded by the spring scale. This procedure was repeated 3 times, and a constant or mean value was noted for each patient to improve the operator's reliability. Further reading was recorded after application of the adhesive cream, powder, and strips in a similar manner according to the manufacturer's instructions over a period of 1 week (Manes et al. 2011).

### 2.12 Statistical Analysis

Descriptive statistics were utilized to explore the frequency, percentage, and mean values of age, gender, and denture weight. The normality of the data was assessed using the Shapiro–Wilk test and histograms to ensure that assumptions underlying parametric tests were met. In the inferential phase of the analysis, the mean values of denture adhesives were calculated, and then compared using analysis of variance (ANOVA) to assess differences across multiple adhesive products. Post‐hoc comparisons were conducted using Tukey's Honestly Significant Difference (HSD) test to determine specific group differences. Additionally, paired *t*‐tests were used to compare the mean values within the groups, particularly to evaluate potential differences between different adhesive products. Furthermore, Pearson correlation analysis was then applied to detect the relationship between denture weight and various adhesive products. A *p*‐value of ≤ 0.05 was considered significant.

## 3 Results

**Figure 6:**
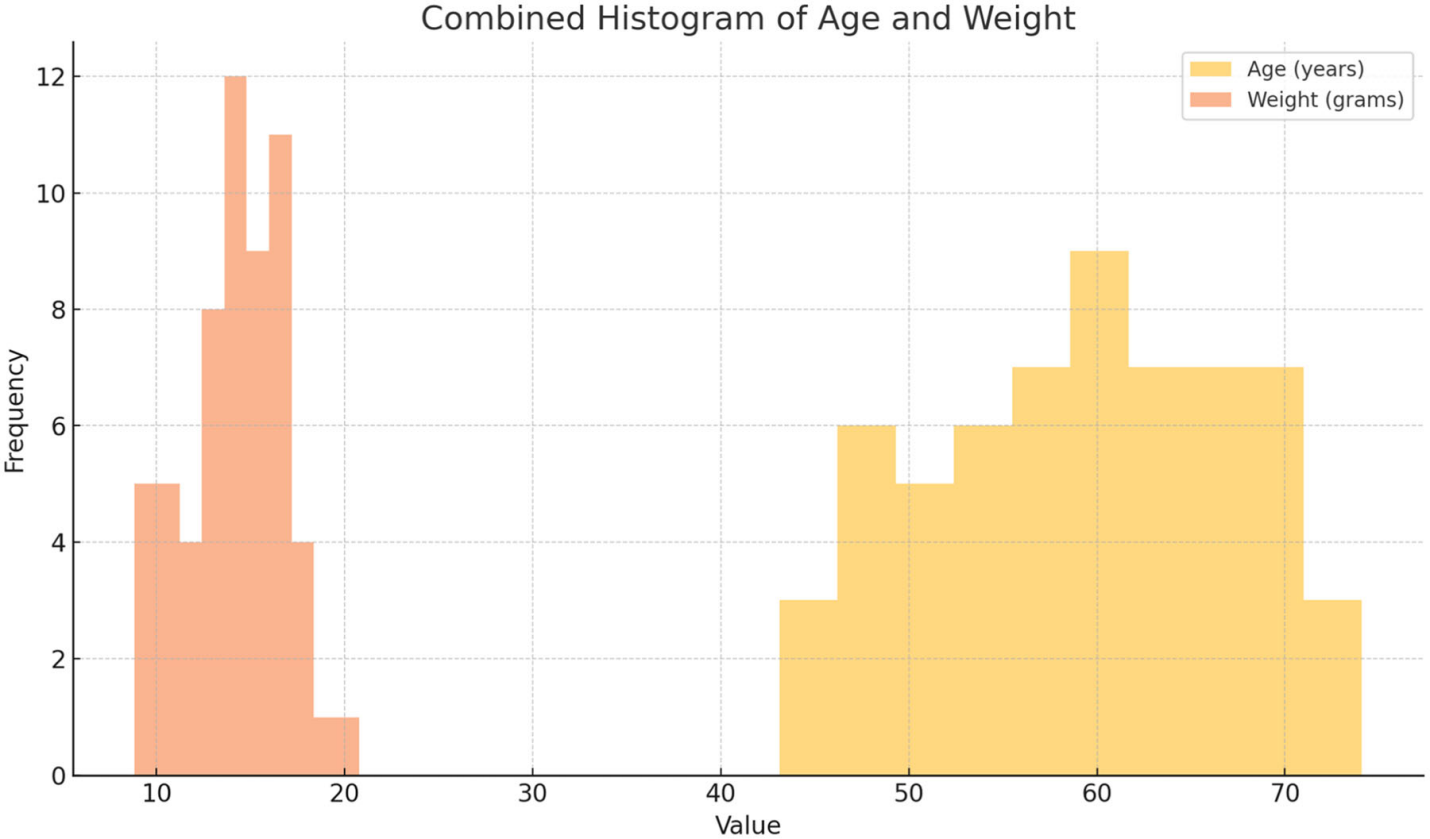
Distribution of participants' age and denture weight.

**Figure 7:**
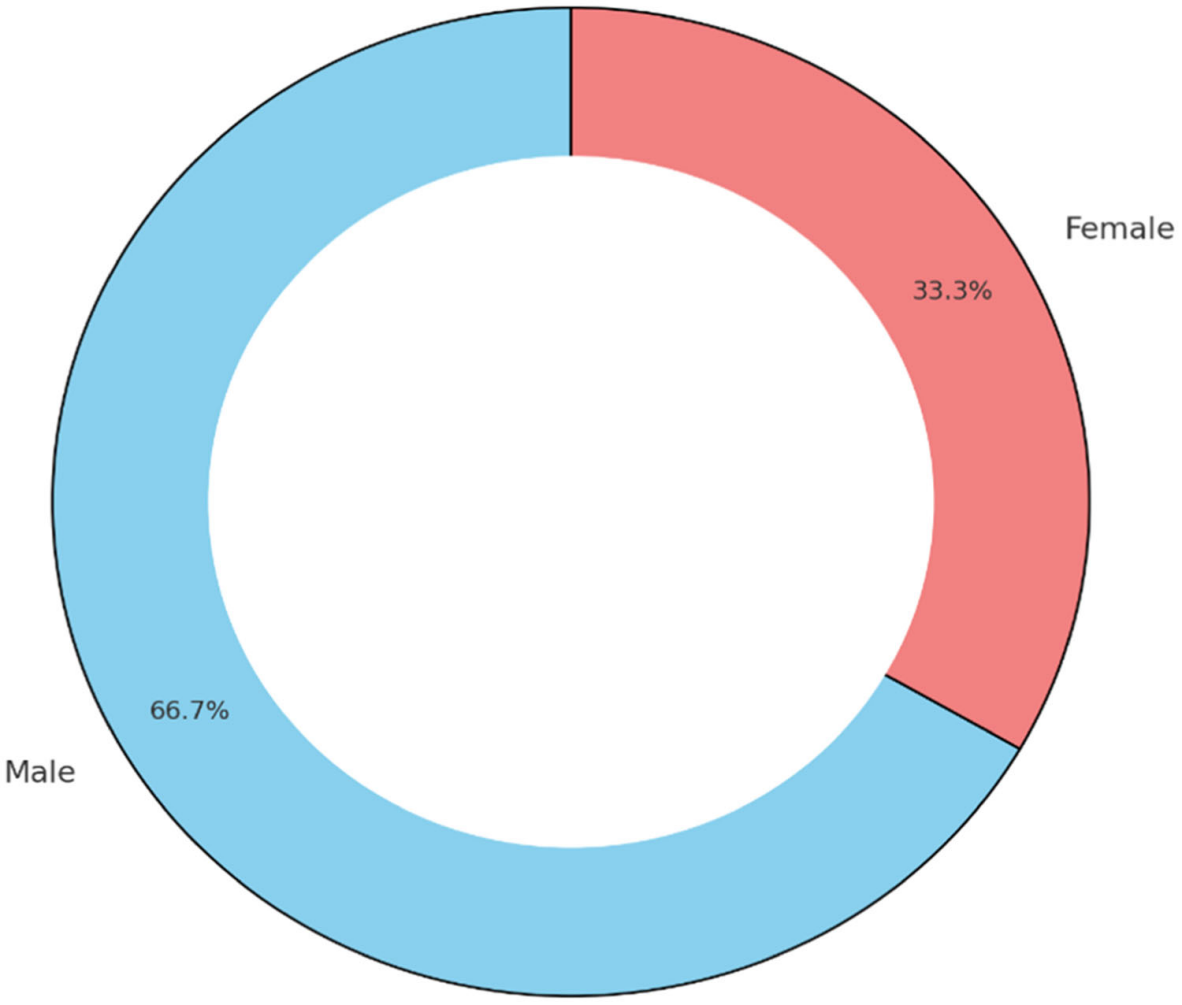
Gender distribution of the study participants.

This study included 60 participants, comprising 40 males (66.7%) and 20 females (33.3%). On an average, participants were 57.63 ± 7.90 years old, whereas the mean weight of the dentures was 14.11 ± 2.24 g (Figures 6 and 7).

For Class 1 ridges, Fittydent cream (2.57 lb) and powder (1.73 lb) showed significant increases compared to no adhesive (0.69 lb), with *p*‐values of 0.001, whereas strips did not show significant increases (0.71 lb, *p* = 0.549). In Class 2, all adhesive cream (1.30 lb), powder (1.06 lb), and strips (1.52 lb)—presented significant differences compared to no adhesive (0.35 lb), with *p*‐values of 0.001. For Class 3, cream (1.22 lb), powder (0.72 lb), and strips (0.72 lb) also displayed significant improvements over no adhesive (0.27 lb), with *p*‐values of 0.001. This indicates the superior retention provided by Fittydent cream and powder across all ridge types, with strips effective in Classes 2 and 3 (Table 3).

**Table 3:** Comparison of retentive strength across ridge classifications with Polygrip adhesive types, *n* = 60 (ANOVA analysis, CI = 95%).

Ridge classification	Retentive strength without adhesives (lb)	Retentive strength with polygrip cream (lb)	Retentive strength with polygrip powder (lb)	Retentive strength with polygrip strips (lb)
Class 1	0.69	2.03	1.07	1.62
Class 2	0.35	1.27	0.77	1.20
Class 3	0.27	1.01	0.49	0.71
*p*‐value	0.001	0.001	0.001	0.004

*Note: p*‐value was significant at ≤ 0.05.

Abbreviation: CI = confidence interval.

Table 4 reveals a significant difference (*p* ≤ 0.05) in retentive strength of fittydent products between the three ridge types. Class 1 showed the highest average baseline strength (0.693 lb) without adhesive, whereas Class 2 (0.407 lb) and Class 3 (0.303 lb) progressively declined. Fittydent cream was the most effective adhesive across all classes. It significantly increases retentive strength compared to control: Class 1 (from 0.69 lb to 2.57 lb), Class 2 (from 0.35 lb to 1.30 lb), and Class 3 (from 0.27 lb to 1.22). Fittydent powder offers some improvement in all classes (Class 1: 1.17, Class 2: 0.913, Class 3: 0.720), but the increase is less substantial than the cream. Fittydent strips showed the least improvement, with Class 1 and Class 3 values (around 0.7) barely exceeding those without adhesives, and Class 2 demonstrated a modest increase (1.088) compared to no adhesive. These findings suggest that among the Fittydent products, the cream form was the most suitable for enhancing denture retention across varying ridge classifications.

**Table 4:** Comparison of retentive strength across ridge classifications with Fittydent adhesive types (ANOVA analysis, CI = 95%), *n* = 60.

Ridge classification	Retentive strength without adhesives (lb)	Retentive strength with Fittydent cream (lb)	Retentive strength with Fittydent powder (lb)	Retentive strength with Fittydent strips (lb)
Class 1	0.69	2.57	1.73	0.71
Class 2	0.35	1.30	1.06	1.52
Class 3	0.27	1.22	0.72	0.72
*p*‐value	0.001	0.001	0.001	0.004

*Note: p*‐value was significant at ≤ 0.05.

Abbreviation: CI = confidence interval.

Table 5 demonstrates significant differences in the fit of different denture adhesives (Fittydent and Polygrip) on Class 1, 2, and 3 ridge types. Fittydent cream and powder, as well as Polygrip cream, powder, and strips, showed significant improvements in retention for Class 1 versus Class 2 and Class 1 versus Class 3 ridge types (*p* < 0.05). Significant differences were also noted between Class 2 and Class 1 for Fittydent powder and strips, and for Polygrip cream, powder, and strips. However, no significant differences (*p* > 0.05) were observed between Class 2 and Class 3 ridges for Fittydent cream and powder, or between Class 1 and Class 2 for Fittydent strips and Polygrip powder.

**Table 5:** Characteristics of post‐hoc Tukey's HSD (*n* = 60).

Variable	Ridge class comparison		Mean Difference	*p*‐value	95% Confidence interval	
					Lower bound	Upper bound
Fittydent cream	Class 1	Class 2	1.26*	0.001	0.805	1.728
		Class 3	1.35*	0.001	0.888	1.811
	Class 2	Class 1	−1.26*	0.001	−1.728	−0.805
		Class 3	0.08	0.902	−0.378	0.544
	Class 3	Class 1	−1.35*	0.001	−1.811	−0.888
		Class 2	−0.08	0.902	−0.544	0.378
Fittydent powder	Class 1	Class 2	0.67*	0.006	0.171	1.168
		Class 3	1.00*	0.001	0.504	1.501
	Class 2	Class 1	−0.67*	0.006	−1.168	−0.171
		Class 3	0.33	0.251	−0.165	0.831
	Class 3	Class 1	−1.003*	0.001	−1.501	−0.504
		Class 2	−0.33	0.251	−0.831	0.165
Fittydent strip	Class 1	Class 2	−0.81*	0.010	−1.455	−0.168
		Class 3	−0.006	1.000	−0.649	0.637
	Class 2	Class 1	0.81*	0.010	0.168	1.455
		Class 3	0.80*	0.011	0.162	1.449
	Class 3	Class 1	0.006	1.000	−0.637	0.649
		Class 2	−0.80*	0.011	−1.449	−0.162
Polygrip cream	Class 1	Class 2	0.75*	0.017	0.115	1.400
		Class 3	1.01*	0.001	0.375	1.660
	Class 2	Class 1	−0.75*	0.017	−1.400	−0.115
		Class 3	0.26	0.596	−0.382	0.902
	Class 3	Class 1	−1.01*	0.001	−1.660	−0.375
		Class 2	−0.26	0.596	−0.902	0.382
Polygrip powder	Class 1	Class 2	0.30*	0.016	0.048	0.555
		Class 3	0.58*	0.001	0.330	0.837
	Class 2	Class 1	−0.30*	0.016	−0.555	−0.048
		Class 3	0.28*	0.026	0.028	0.535
	Class 3	Class 1	−0.58*	0.001	−0.837	−0.330
		Class 2	−0.28*	0.026	−0.535	−0.028
Polygrip strip	Class 1	Class 2	0.41	0.154	−0.115	0.939
		Class 3	0.90*	0.001	0.379	1.434
	Class 2	Class 1	−0.41	0.154	−0.939	0.115
		Class 3	0.49	0.070	−0.032	1.022
	Class 3	Class 1	−0.90*	0.001	−1.434	−0.379
		Class 2	−0.49	0.070	−1.022	0.032

*Note: p*‐value was significant at ≤ 0.05.

The comparison of ridge types with fittydent denture adhesive consistencies showed statistically significant differences from the control group. However, for Fittydent strips in the Class 1 ridge type, the difference was not statistically significant (*p* = 0.549) (Table 6).

**Table 6:** Comparison of Fittydent denture adhesive retentive strength in the experimental and control groups in different ridge types (*n* = 60).

Ridge classification	Control vs. Fittydent cream (*p*‐value)	Control vs. Fittydent powder (*p*‐value)	Control vs. Fittydent strips (*p*‐value)
Class 1	0.001*	0.001*	0.549
Class 2	0.001*	0.001*	0.001*
Class 3	0.001*	0.001*	0.001*

Abbreviation: CI = confidence interval.

* The *p*‐value was significant at ≤ 0.05

Table 7 shows a statistically significant difference (*p* = 0.001) in the mean retentive strength values between the experimental group using Polygrip products and the control group across all ridge types. This suggests that Polygrip products significantly improved denture retention compared to the control group, regardless of ridge classification.

**Table 7:** Comparison of Polygrip denture adhesive retentive strength in the experimental and control groups in different ridge types (*n* = 60).

Ridge classification	Control vs. polygrip cream (*p*‐value)	Control vs. polygrip powder (*p*‐value)	Control vs. polygrip strips (*p*‐value)
Class 1	0.001*	0.001*	0.001*
Class 2	0.001*	0.001*	0.001*
Class 3	0.001*	0.001*	0.001*

* The *p*‐value was significant at ≤ 0.05.

There was a weak negative correlation with Fittydent strip (rho = −0.332), which was statistically significant at (*p* = 0.010), indicating a potential relationship between adhesive efficacy and denture weight, specifying its improved efficacy in denture retention. However, no significant correlations were found between denture weight and other adhesive types, including Fittydent cream (0.017, *p* = 0.899), Fittydent powder (0.081, *p* = 0.539), Polygrip cream (−0.160, *p* = 0.221), Polygrip powder (−0.065, *p* = 0.619), and Polygrip strip (−0.182, *p* = 0.163), suggesting that there is no statistically significant relationship between the use of these adhesives and denture weight when used without adhesive. The retentive strength of the denture does not appear to be significantly affected by the weight of the denture (Table 8).

**Table 8:** Correlation between denture weight and various adhesive types.

Variable		Without adhesive	Fittydent cream	Fittydent powder	Fittydent strip	Polygrip cream	Polygrip powder	Polygrip strip
Weight	Pearson correlation	−0.216	0.017	0.081	−0.332*	−0.160	−0.065	−0.182
	*p*‐value	0.097	0.899	0.539	0.010	0.221	0.619	0.163
	*N*	60	60	60	60	60	60	60

* Correlation is significant at the 0.05 level (2‐tailed). *N*, number of dentures.

## 4 Discussion

This study highlights the importance of selecting appropriate denture adhesive products according to the residual alveolar ridge morphology. The hypothesis regarding the effectiveness of denture adhesives across different ridge types was supported, as evidenced by the significant variations in retentive strength observed among ridge varieties with different adhesive types. However, the prediction regarding the correlation between mandibular denture weight and adhesive efficacy was partially accepted, specifically for Fittydent strips. Therefore, the hypothesis regarding the effectiveness of denture adhesives across different ridge types was accepted, whereas the prediction regarding the correlation between denture weight and adhesive efficacy was rejected. In this study, Polygrip cream overall demonstrated superior efficacy in enhancing denture retention, better than adhesive powder and strips. Similarly, in another study, cream‐based adhesives were reported to have better retention and comfort compared to other forms due to their ability to spread evenly over the denture base and oral mucosa. Fittydent cream showed promising results, although slightly less effective than Polygrip cream (Sipayung and Ariyani 2020). These findings highlight the need for clinicians to consider the specific characteristics of adhesive types when prescribing them to patients with different alveolar ridge types.

This study revealed significant differences in retentive strength across different ridge types studied, with Class 1 showing the highest baseline strength without adhesives. This observation suggests a potential relationship between ridge morphology and denture fit, wherein patients with mild ridge resorption may experience better denture stability. However, even in Class 1 ridges, the application of denture adhesives significantly improved retention, emphasizing the importance of adhesive use regardless of ridge type. Conversely, Class 3 ridges demonstrated the lowest baseline retentive strength, indicating a critical need for additional retention strategies beyond conventional denture fabrication techniques.

The current study compared the efficacy of two commonly used adhesive brands, Fittydent and Polygrip, across various ridge classifications. Although both brands demonstrated effectiveness in improving denture retention, Polygrip cream consistently showed better performance than Fittydent. This finding suggests that factors such as adhesive composition, application method, and adhesive base play crucial roles in determining adhesive efficacy. Additionally, the study identified Fittydent powder and strips as viable alternatives, although with slightly lower efficacy compared to their cream type. This study supports previous research demonstrating the significant enhancement of denture retention with adhesive use, particularly with Polygrip cream; notable variations exist in the specific performance of adhesive formulations across ridge morphologies. This aligns with a similar study, which similarly emphasized the importance of personalized adhesive selection based on individual patient characteristics (Yamaguchi et al. 2023; Silva et al. 2020). However, discrepancies emerge regarding the optimal adhesive type for specific ridge classifications, with some studies advocating for powder formulations in well‐formed ridges (Malhotra et al. 2013), whereas others emphasize the efficacy of cream and strip formulations in various ridge conditions (Sipayung and Ariyani 2020; Swelem et al. 2025). On the contrary, a study reported that the use of denture adhesive, whether in paste, powder, or cushion form, effectively increased the retention of mandibular dentures, with no significant variance observed among the different adhesive types (Teama et al. 2021). Such discrepancies may result from differences in study methodologies, participant demographics, and adhesive application techniques, highlighting the need for further investigation to clarify the most effective adhesive strategies for diverse patient populations.

Furthermore, we revealed a weak negative correlation between denture weight and adhesive efficacy, particularly notable with Fittydent strips. This challenges conventional assumptions and highlights the complexity of factors influencing denture retention. Comparison of our findings with the existing literature highlights inconsistencies in the relationship between denture weight and retention reported in previous studies (Akl and Stendahl 2022; Ohkubo and Hosoi 1999). The disparities in the relationship between denture weight and retention reported in our study compared to the previous literature could stem from variations in study methodologies, participant demographics, and adhesive application techniques. Additionally, differences in denture materials, design, and fit may contribute to conflicting findings across studies. Furthermore, variations in oral anatomy, such as ridge morphology and tissue resilience, among study populations could influence the interaction between denture weight and adhesive efficacy. These potential factors highlight the complexity of denture retention dynamics and highlight the need for further research to clarify the underlying mechanisms driving these discrepancies.

The findings of this study have significant implications for clinical practice, emphasizing the importance of personalized denture adhesive selection based on individual patient characteristics. Prosthodontists should consider factors such as ridge morphology, patient preferences, and adhesive properties when recommending denture adhesives to enhance patient comfort and satisfaction. Moreover, regular follow‐up assessments and adjustments may be necessary to ensure optimal denture fit and retention, particularly in individuals with severe ridge resorption or fluctuating oral conditions.

The variation in the amount of denture adhesive applied by patients, ranging from 0.5 to 3 cm, in this study is a notable finding that warrants discussion. This amount of adhesive may be influenced by multiple factors, including differences in residual ridge morphology, patient experience with adhesives, and individual perception of the required amount for optimal retention. Patients with severe ridge resorption or ill‐fitting dentures may apply larger quantities, whereas those with well‐adapted prostheses may use minimal amounts. Despite this variation, the study findings suggest that even small amounts of adhesive can enhance retention and stability, supporting its role as an adjunct in complete denture wearers. However, excessive use may lead to patient discomfort, altered occlusion, or difficulty in cleaning, emphasizing the need for professional guidance on appropriate application techniques.

It is essential to acknowledge the limitations of this study, including its small sample size and short‐term follow‐up period, and also the use of semi‐anatomic teeth for all ridge types, which may not fully represent the variability in tooth forms recommended for specific ridge classifications in clinical practice. The decision to use semi‐anatomic teeth for all ridge types in the study was made to maintain consistency and simplify the denture fabrication process, ensuring uniformity in the occlusal scheme and tooth anatomy. This approach was chosen to focus on evaluating the comparative effectiveness of different denture adhesives without introducing variability from differing tooth forms, thereby enhancing the study reliability and practicality within its defined scope. Future research with larger sample sizes and longer follow‐up durations is warranted to validate the findings of this study and explore additional factors influencing denture adhesive efficacy, such as patient compliance, oral hygiene, and denture maintenance practices. Furthermore, comparative studies evaluating the cost‐effectiveness and long‐term outcomes of different adhesive products are needed to inform evidence‐based clinical decision‐making.

This study benefits from several strengths that enhance the robustness and applicability of its findings. First, the utilization of an interventional design ensures a high level of internal validity by minimizing bias and confounding variables. Crossover trials minimize confounding variables and enhance the precision of treatment effect estimates. It also reduces interindividual variability, thereby increasing the statistical power to detect treatment effects. Additionally, the inclusion of participants with diverse ridge morphologies and denture weights enhances the generalizability of the study results to a broader population of complete denture wearers. Moreover, the standardized protocols used for denture fabrication and adhesive application enhance the consistency and reliability of the study outcomes. The comprehensive assessment of multiple adhesive products provides clinicians with valuable information to make informed decisions regarding adhesive selection for their patients.

The study emphasizes the importance of personalized adhesive selection tailored to individual patient needs. Further analysis into long‐term outcomes and patient‐reported satisfaction metrics will be imperative to fully comprehend the clinical implications of these findings and to continually improve the quality of care provided to denture wearers.

## 5 Conclusion

The findings of this study highlight the significance of personalized denture adhesive selection based on ridge classification and denture weight to enhance retention and comfort among denture wearers. Polygrip cream emerged as the most effective adhesive across all ridge classifications, followed by Fittydent cream, whereas Fittydent powder and strips showed comparatively lesser efficacy. These findings provide valuable insights for dental practitioners in optimizing denture adhesive selection based on both ridge types and denture weight to improve patient outcomes and satisfaction.

## Author Contributions

*Conceptualization*: Naseer Ahmed, Maria Shakoor Abbasi, Asra Salahuddin, Lareb Tariq, Fahim Vohra, Artak Heboyan, Gotam Das, Ghazala Suleman, and Sarrah Sira. *Methodology*: Naseer Ahmed, Maria Shakoor Abbasi, Asra Salahuddin, Fahim Vohra, and Lareb Tariq. *Software:* Artak Heboyan, Naseer Ahmed, Asra Salahuddin, and Gotam Das. *Validation*: Naseer Ahmed, Maria Shakoor Abbasi, and Asra Salahuddin. *Formal analysis*: Naseer Ahmed and Asra Salahuddin. *Investigation*: Naseer Ahmed, Maria Shakoor Abbasi, Asra Salahuddin, and Sarrah Sira. *Resources*: Artak Heboyan, Ghazala Suleman, Gotam Das, Fahim Vohra, and Naseer Ahmed. *Data curation*: Lareb Tariq, Asra Salahuddin, Maria Shakoor Abbasi, Sarrah Sira, and Naseer Ahmed. *Writing – original draft preparation*: Naseer Ahmed, Asra Salahuddin, Maria Shakoor Abbasi, Gotam Das, Artak Heboyan, *Writing – review and editing*: Naseer Ahmed, Artak Heboyan, Ghazala Suleman, Gotam Das, and Sarrah Sira. *Visualization*: Maria Shakoor Abbasi, Naseer Ahmed, Sarrah Sira, Asra Salahuddin, and Fahim Vohra. *Supervision*: Naseer Ahmed, Maria Shakoor Abbasi, and Fahim Vohra. *Project administration*: Maria Shakoor Abbasi, Naseer Ahmed, Asra Salahuddin, Lareb Tariq, and Sarrah Sira.

## Ethics Statement

Ethical approval was obtained from the Ethical Review Committee of the Altamash Institute of Dental Medicine (AIDM/ERC/01/2021/03). Informed consent was obtained from all subjects involved in this study.

## Consent

Consent was obtained from all participants for publication.

## Conflicts of Interest

The authors declare no conflicts of interest.

## Data Availability

The data that support the findings of this study are available from the corresponding author upon reasonable request.
